# Survival Prediction of Patients Who Were Terminally Ill Using the EORTC QLQ-C15-PAL Scores and Laboratory Test Values

**DOI:** 10.1089/pmr.2023.0015

**Published:** 2023-08-01

**Authors:** Chikako Matsumura, Nanako Koyama, Kaho Okuno, Nobuhiko Nakamura, Morito Sako, Hideo Kurosawa, Takehisa Nomura, Yuki Eguchi, Kazuki Ohba, Yoshitaka Yano

**Affiliations:** ^1^ Education and Research Center for Clinical Pharmacy, Kyoto Pharmaceutical University, Kyoto, Japan.; ^2^ Department of Pharmacy, Yodogawa Christian Hospital, Osaka, Japan.; ^3^ Department of Pharmacy, Tachibana Medical Corporation Higashisumiyoshi-Morimoto Hospital, Osaka, Japan.; ^4^ Palliative Care Unit, Tachibana Medical Corporation Higashisumiyoshi-Morimoto Hospital, Osaka, Japan.; ^5^ Department of Palliative Care, Tachibana Medical Corporation Higashisumiyoshi-Morimoto Hospital, Osaka, Japan.

**Keywords:** palliative care, quality of life, survival prediction, symptom cluster, symptom score, terminally ill cancer patient

## Abstract

**Background:**

Prognostics for patients with cancer is especially important for the supportive care of those who are terminally ill. We previously found that symptom scores as patient-reported outcomes (PROs)—such as dyspnea and fatigue scores—some biochemical parameters, the palliative performance scale (PPS) scores, and symptom clusters were useful prognostic factors; however, the predictability of a prognosis based on these factors remains unclear.

**Objective:**

To identify appropriate three-week survival predictive factor(s), in terms of performance, in patients who were terminally ill.

**Design:**

We collected symptom scores as PROs using the Japanese version of the European Organization for Research and Treatment of Cancer Quality of Life Questionnaire Core 15 Palliative Care (EORTC QLQ-C15-PAL).

**Setting/Subjects:**

We used data from terminally ill patients with cancer who were hospitalized at the palliative care unit of the Higashisumiyoshi-Morimoto Hospital (Osaka, Japan) from June 2018 to December 2019 (*n* = 130), as well as additional data obtained from the same clinical study from January to March 2020 (*n* = 31).

**Measurements:**

To evaluate predictive performance, indices such as sensitivity, specificity, positive predictive value, negative predictive value, and overall accuracy were calculated.

**Results:**

We found that the presence of a symptom cluster showed high sensitivity but low specificity and that a higher PPS value (>30) showed high specificity but low sensitivity, suggesting that these factors could provide relevant information for survival prognosis (less than or equal to three weeks).

**Conclusion:**

Symptom clusters obtained from patients is important for effective supportive care of those who are terminally ill.

## Introduction

The availability of accurate prognostic information for patients with advanced cancer is of great importance not only for timely and appropriate advance care planning but also to incorporate advanced health care decision making.^1–3^ A previous study that included 130 patients with cancer, hospitalized in a palliative care unit, evaluated not only symptom scores as patient-reported outcomes (PROs)—such as dyspnea and fatigue scores—but also levels of biochemical parameters—such as the C-reactive protein (CRP), albumin (Alb), neutrophil–lymphocyte ratio,^4^ and the Palliative Performance Scale (PPS)—and the existence of symptom clusters.^5^ A symptom cluster is defined as two or more symptoms that are related to each other and occur together.^6^ These items act as important independent prognostic factors, which can result in poor PPS scores (≤30). Furthermore, the presence of a symptom cluster that included dyspnea, appetite loss, fatigue, and nausea was identified as an indicator of poor prognosis.^4,5^

Although a previous study detected significant risk factors for poor survival and determined the cutoff values of the measures or scores, the predictability of prognosis based on these factors remains unclear. In this study, we focus on the prediction of a three-week survival. By examining the sensitivity, specificity, positive predictive value (PPV), negative predictive value (NPV), and overall accuracy of these clinical factors, we aimed to identify appropriate factor(s) to evaluate a three-week survival predictive performance of these prognostic indicators in terminally ill patients with cancer.

## Materials and Methods

### Patients

All data used in this study were obtained from a previous clinical observational study^4,5^ conducted at Tachibana Medical Corporation Higashisumiyoshi-Morimoto Hospital (Osaka, Japan). Part of the data collected from June 2018 to December 2019, which were used in these previously published data analyses^4,5^ (*n* = 130), were considered as a training dataset in the present analysis. We collected new additional data—obtained from the same clinical study at a different period from that of the training data—on terminally ill patients with cancer who were hospitalized in the same palliative care unit from January to March 2020 (*n* = 31). These data were used as a test dataset for validation. As per the hospital policy of providing the best care support, the palliative care unit is intended for patients who require total pain relief and whose families wish for them to be hospitalized in the unit.

The additional data were obtained by using common procedures based on a previous study.^4^ We collected symptom scores at the time of hospitalization as PROs using the Japanese version of the European Organization for Research and Treatment of Cancer Quality of Life Questionnaire Core 15 Palliative Care (EORTC QLQ-C15-PAL), with permission from the developer. The inclusion criteria for this study were those patients who could answer the questionnaires. The QLQ-C15-PAL, a self-report tool with 10 domains of 15 items evaluated patients' quality of life (QOL).

Analyses were conducted using the responses for the two functional domains—physical functioning (three items) and emotional functioning (two items)—and seven symptom domains—dyspnea (one item), pain (two items), insomnia (one item), appetite loss (one item), constipation (one item), fatigue (two items), and nausea (one item)—rated on a 4-point Likert scale (1 = not at all, 2 = a little, 3 = quite a bit, and 4 = very much), with QOL being rated on 7-point scale. In this analysis, all scales of the 10 items, which ranged from 0 to 100, were linearly transformed according to a previous publication.^7,8^ Baseline data from the new patients, which included age, gender, type of cancer, presence or absence of metastasis, values of inflammatory biomarkers (CRP and Alb), and PPS, were collected from their medical records at the time of hospitalization. In addition, all patients were followed up until death or discharge during the study period.

The study was conducted in accordance with the Declaration of Helsinki and Ethical Guidelines for Epidemiology Research. It was also approved by both the Ethics Committees of the Higashisumiyoshi-Morimoto Hospital (on May 15, 2018) and Kyoto Pharmaceutical University (No. 20–18-02, 2021), to which the authors are affiliated. All patients provided verbal informed consent to use their data in the study.

### Statistical analysis

The patients' characteristics in the training and test datasets were summarized and compared. Especially for the QLQ-C15-PAL scores, the average values were compared between the datasets for the transformed 10 items. The cutoff values were already determined in a previous study using a receiver operating characteristic (ROC) analysis of the training data for “dyspnea” and “fatigue” in the QLQ-C15-PAL, CRP, Alb, and PPS.^4,5^ According to these previous results,^4^ the factors that significantly predicted prognosis, which was defined as a three-week survival, were the scores for “dyspnea” and “fatigue” in the QLQ-C15-PAL, CRP, Alb, PPS, and the presence of a symptom cluster. Previous statistical analysis^5^ defined the presence of a symptom cluster as the presence of at least three items among dyspnea (one item), appetite loss (one item), fatigue (two items), and nausea (one item) through the QLQ-C15-PAL. In both the training and test datasets, the actual survival data were categorized for a three-week survival.

In the previous study,^4^ results of the ROC analysis of the statistically significant variables to detect prognostic risk of less than three weeks were summarized, and the cutoff values were estimated at 66.67 (dyspnea), 66.67 (fatigue), 3.0 mg/dL (CRP), and 2.5 g/dL (Alb). In addition, the predicted survival was categorized by the presence and absence of each factor, which resulted in a 2 × 2 contingency table. Finally, sensitivity (true positive rate), specificity (true negative rate), PPV, NPV, and overall accuracy (number of correct predictions divided by the number of total predictions) at predicting a three-week survival rate were calculated to evaluate the factors' predictability.

The censored data, for which a survival time could not be obtained, were excluded for the survival prediction. Sensitivity and specificity for the factors in the training dataset were estimated when the cutoff values were evaluated.^4^ In this study, we additionally estimated the predictive performance including a new factor “symptom cluster”^5^ using other indices such as PPV, NPV, and overall accuracy.

Data were summarized using Microsoft Excel^®^, and all statistical analyses were performed using the Bell Curve for Excel 2.15^®^ (Social Survey Research Information Co., Ltd., Tokyo, Japan). Statistical tests, such as an unpaired *t* test, Mann–Whitney *U* test, and chi-squared test, were conducted as appropriate. The details of the test methods are given in the footnotes of the tables.

## Results

For this study, we obtained new data from 31 patients who completed the QLQ-C15-PAL questionnaire at the time of hospitalization. Table 1 summarizes the patients' characteristics in the training and test datasets. There were no statistically significant differences in age, gender, PPS, type of cancer, ratio of the presence of metastasis, inflammatory biomarkers, or survival time between the groups. From these results, we assumed that the data in both sets were obtained from the same patient population.

**Table 1.** Patients' Characteristics

	Training dataset	Test dataset	***p***
Total No. of patients	130	31	
Age (years), median (min.–max.)	74, 32–97	72, 40–88	0.10^*^
Gender (male/female)	71 (54.6)/59 (45.4)	15 (48.4)/16 (51.6)	0.53^**^
PPS			
≥70	20 (15.4)	3 (9.7)	0.60^**^
40–60	74 (56.9)	19 (61.3)	
≤30	29 (22.3)	9 (29.0)	
Unknown	7 (5.4)	0 (0)	
Type of cancer			
Lung	31 (23.8)	5 (16.1)	0.26^**^
Colorectal	26 (20.0)	7 (22.6)	
Pancreatic	12 (9.2)	4 (12.9)	
Gastric	7 (5.4)	3 (9.7)	
Liver	7 (5.4)	2 (6.5)	
Breast	5 (3.8)	1 (3.2)	
Esophageal	5 (3.8)	1 (3.2)	
Ovarian	4 (3.1)	0 (0)	
Uterine	4 (3.1)	2 (6.5)	
Prostate	3 (2.3)	1 (3.2)	
Other	26 (20.0)	3 (9.7)	
Unknown	0 (0)	2 (6.5)	
Metastasis (no/yes)	16 (12.3)/114 (87.7)	2 (6.5)/29 (93.5)	0.35^**^
Inflammatory biomarkers (median, min.–max.)	*n* = 126	*n* = 27	
CRP (mg/dL)	4.0 (<0.1–32.1)	6.4 (0.7–26.4)	0.05^*^
Alb (g/dL)	2.6 (1.2–3.9)	2.4 (1.6–3.6)	0.22^*^
Survival time, median (No. of patients who died during the study period)			
	18 (*n* = 109)	18 (*n* = 27)	0.41^***^

Numbers in parentheses are the percentage to the total numbers of patients unless stated otherwise.

^*^ Unpaired two-sided *t* test; ^**^Chi-squared test; ^***^Mann–Whitney *U* test.

Alb, albumin; CRP, C-reactive protein; PPS, Palliative Performance Scale.

Table 2 summarizes the QLQ-C15-PAL scores for the datasets at the time of hospitalization. Owing to some missing data, the actual number of patients whose data were obtained is reflected in Table 2. No statistically significant differences were observed in any of the scores. As previously mentioned, higher physical and emotional functioning scores indicated a “better” patient condition. In contrast, higher scores for other symptoms indicated a “worse” patient condition. Similar to previous results from the training set, physical functioning, appetite loss, and fatigue scores indicated relatively worse QOL in patients in the test dataset.

**Table 2.** Summary of the QLQ-C15-PAL Scores at the Time of Hospitalization

QLQ-C15-PAL (median, mean [±SD])	***n***	Training dataset (***n*** = 130)	***n***	Test dataset (***n*** = 31)	***p***
Physical functioning	125	33.3, 37.0 ± 25.1	31	46.7, 40.0 ± 18.7	0.21
Emotional functioning	129	66.7, 63.2 ± 28.3	30	66.7, 59.4 ± 32.5	0.66
Dyspnea	130	33.3, 39.5 ± 34.4	31	33.3, 45.2 ± 38.1	0.49
Pain	129	50.0, 47.3 ± 34.0	31	33.3, 46.2 ± 37.4	0.79
Insomnia	130	33.3, 40.0 ± 36.0	31	33.3, 41.9 ± 40.3	0.87
Appetite loss	130	66.7, 57.7 ± 38.4	30	66.7, 60.0 ± 38.6	0.79
Constipation	128	33.3, 37.0 ± 35.3	28	33.3, 44.8 ± 43.0	0.46
Fatigue	129	66.7, 63.8 ± 29.7	31	88.9, 74.6 ± 26.6	0.12
Nausea/vomiting	130	0.0, 18.7 ± 29.6	31	0.0, 23.1 ± 37.4	0.86
QOL	124	50.0, 38.7 ± 27.1	30	33.3, 35.6 ± 28.6	0.43

In the QLQ-C15-PAL, patients rated their symptoms on a 4-point scale (1 = not at all, 2 = a little, 3 = quite a bit, and 4 = very much) for the two functional domains and seven symptom domains, and a 7-point scale (range: 1 = very poor to 7 = excellent) for their overall QOL. All the scale scores were linearly transformed, and the resultant scores ranged from 0 to 100. *p*-values were determined using a nonparametric Mann–Whitney *U* test.

QLQ-C15-PAL, European Organization for Research and Treatment of Cancer Quality of Life Questionnaire Core 15 Palliative Care; QOL, quality of life.

Table 3 shows the sensitivity, specificity, PPV, NPV, and overall accuracy for each risk factor. Missing data for each factor caused the values to vary; the actual sample size analyzed for each factor can be found in Table 3. The cutoff values of each factor are presented in the footnote of Table 3. As the number of patients in the test dataset was small, we evaluated predictability not only in the test dataset but also in the training dataset. The presence of a symptom cluster showed relatively high sensitivity values (>80%) but low specificity. However, the sensitivity of other items was not necessarily high, which suggested that the presence of a symptom cluster is a possible index to predict poor survival prognosis (within three weeks) in terminally ill patients. In contrast, the PPS showed higher specificity values (>80%) in both datasets, but low sensitivity. This suggested that a PPS value >30 could provide relevant information for predicting survival of more than three weeks. Values of other items were not good enough for predicting survival.

**Table 3.** Prediction Accuracy of Shorter Prognosis for Each Risk Factor in the Training and Test Datasets Using the Cutoff Values Obtained in a Previous Study^4^ (Unit [%])

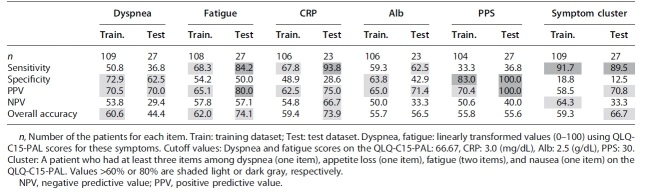

## Discussion

We examined the performance of some prognostic indicators to predict a three-week survival in hospitalized terminally ill patients with cancer. We found that the presence of a symptom cluster could provide relevant information for a poor survival prognosis. The symptom cluster was defined as the condition “when two or more of the symptoms such as dyspnea, appetite loss, fatigue, and nausea were present among the QLQ-C15-PAL scores.”^5^ When two or more of these symptoms exist, the probability of correctly predicting a patient's prognosis was found to have high sensitivity but very poor specificity in both the training and test datasets. The sensitivity in cases where only dyspnea or fatigue was present was not better than that in cases of symptom clusters.

Various distress symptoms occur in terminally ill patients. Our results suggest the importance of detecting symptom clusters by paying attention to patients' complaints of distress symptoms to provide effective palliative care by predicting their survival. Several studies have reported a significant association between PPS scores and survival among palliative care patients with advanced cancer.^9^ We found that the PPS scores, which is assessed by medical professionals, showed good specificity but poor sensitivity suggesting that PPS would not necessarily be an indicator to predict worse survival prognosis (less than or equal to three weeks) in terminally ill patients with cancer. Higher PPS value (≥30) could provide relevant information of better survival prognosis.

Several previous studies reported that some symptom scores obtained as PROs were significantly associated with survival data.^4,10–16^ Furthermore, these symptom scores were identified as independent prognostic factors that influenced survival in patients with advanced cancer.^4,10–16^ In these studies, the symptom burden was a good indicator of poor prognosis in terminally ill patients with cancer,^16^ and dyspnea and fatigue were important symptoms for predicting shorter survival time.^17^

In this study, our results indicate that some symptom scores and their combination as a symptom cluster are useful, as are other widely used parameters such as the palliative prognosis (PaP) score and palliative prognostic index, to predict patients' survival prognosis.^17,18^ For daily supportive care, especially when clinical laboratory test data are unavailable, our results suggest that using symptom scores by PROs may be useful to assess patients' needs. A previous study^19^ found some biases between PROs and clinicians' reported outcomes (CROs). Both PROs and CROs should be applied appropriately in clinical settings, as PROs serve as a good measure to evaluate patients' symptom burdens.

This study has some limitations. First, the number of patients in the test dataset was limited. Second, the data were obtained from a single hospital, which may limit the generalizability of our results. There may be a bias when applying the current results to other patients and, thus, these results should be treated cautiously.

## Conclusion

We aimed to find the most appropriate factors for predicting a three-week survival in hospitalized terminally ill patients. We found that the presence of a symptom cluster could provide relevant information to predict poor survival rates. We also found that a higher PPS value (>30) could be a possible factor for predicting better survival rates (more than three weeks). We conclude that detecting symptom clusters among various distress factors obtained as PROs could be useful for the supportive care of terminally ill patients.

## Abbreviations Used

Alb: albumin
CROs: clinicians' reported outcomes
CRP: C-reactive protein
EORTC QLQ-C15-PAL: European Organization for Research and Treatment of Cancer Quality of Life Questionnaire Core 15 Palliative Care
NPV: negative predictive value
PaP: palliative prognosis
PROs: patient reported outcomes
PPS: palliative performance scale
PPV: positive predictive value
QOL: quality of life
ROC: receiver operating characteristic
